# A typical case of bilateral cataract with corneal arcus senilis

**DOI:** 10.11604/pamj.2025.52.93.49281

**Published:** 2025-11-03

**Authors:** Amol Madhav Deshpande, Mayuri Amol Deshpande

**Affiliations:** 1 Department of Rachana Sharir, Mahatma Gandhi Ayurved College Hospital and Research Centre, Datta Meghe Institute of Higher Education and Research (Deemed to be University), Salod (H), Wardha, Maharashtra, India,; 2 Department of Kayachikitsa, Mahatma Gandhi Ayurved College Hospital and Research Centre, Datta Meghe Institute of Higher Education and Research (Deemed to be University), Salod (H), Wardha, Maharashtra, India

**Keywords:** Bilateral cataract, corneal arcus senilis, mature cataract

## Image in medicine

Cataract is a common problem in tropical areas and countries like India. In cataract, there is opacity in the lens, causing progressive diminishing in vision and troubling day-to-day life. Cataract is the leading cause of blindness worldwide. The minor surgery, known as extraction, is beneficial. Here is a case of mature bilateral cataract. A 75-year-old woman came to the outpatient department of Mahatma Gandhi Ayurved College Hospital and Research Centre, Salod (H), Wardha, Maharashtra, India. She had blurred vision, which was diminishing day by day. The cataracts were mature enough that they could be seen with the naked eye without the assistance of a torch. Along with this finding, there were senile rings surrounding both corneas, which is known as corneal arcus senilis. The patient was referred to an ophthalmologist for further procedure.

**Figure 1:**
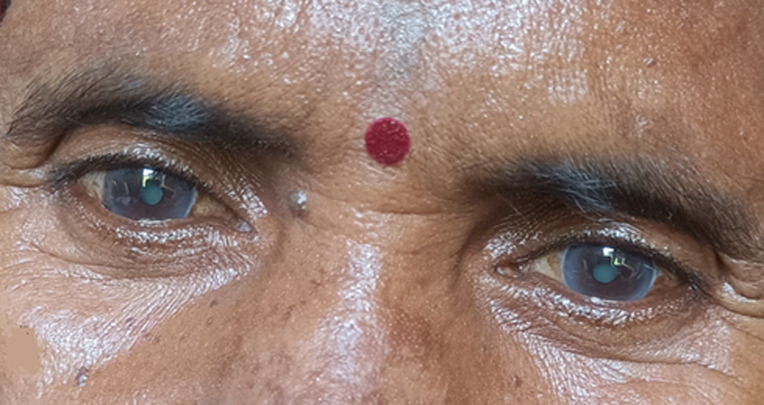
bilateral cataract with corneal arcus senilis

